# Supplementary material for: Endosome motility controls light-responsive reproductive development and secondary metabolite production in Aspergillus
Source: bioRxiv. 2026 Mar 4:2026.03.03.708097. Preprint. [Version 1] doi: 10.64898/2026.03.03.708097 (PMC12991151; doi:10.64898/2026.03.03.708097)
Supplement: 1 [file NIHPP2026.03.03.708097v1-supplement-1.pdf]

bioRxiv preprint doi: https://doi.org/10.64898/2026.03.03.708097; this version posted March 4, 2026. The copyright holder for this preprint (which was not certified by peer review) is the author/funder, who has granted bioRxiv a license to display the preprint in perpetuity. It is made available under aCC-BY 4.0 International license.

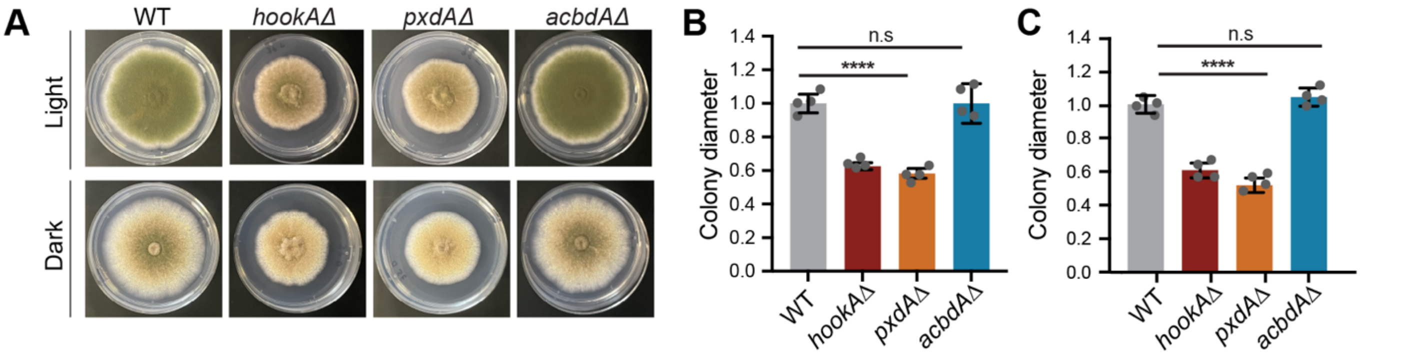

Figure S1 (related to figure 1). Loss of pxdA and hookA reduces radial growth in Aspergillus nidulans (A) Images of agar plates for WT, *hookAΔ*, *pxdAΔ*, and *acbdAΔ* strains grown in light (top) or dark (bottom). **(B and C)** Quantification of radial growth in light (B) and dark (C) condition, normalized to WT strain. Data are represented as mean ± SD (n = 4 biological replicates).

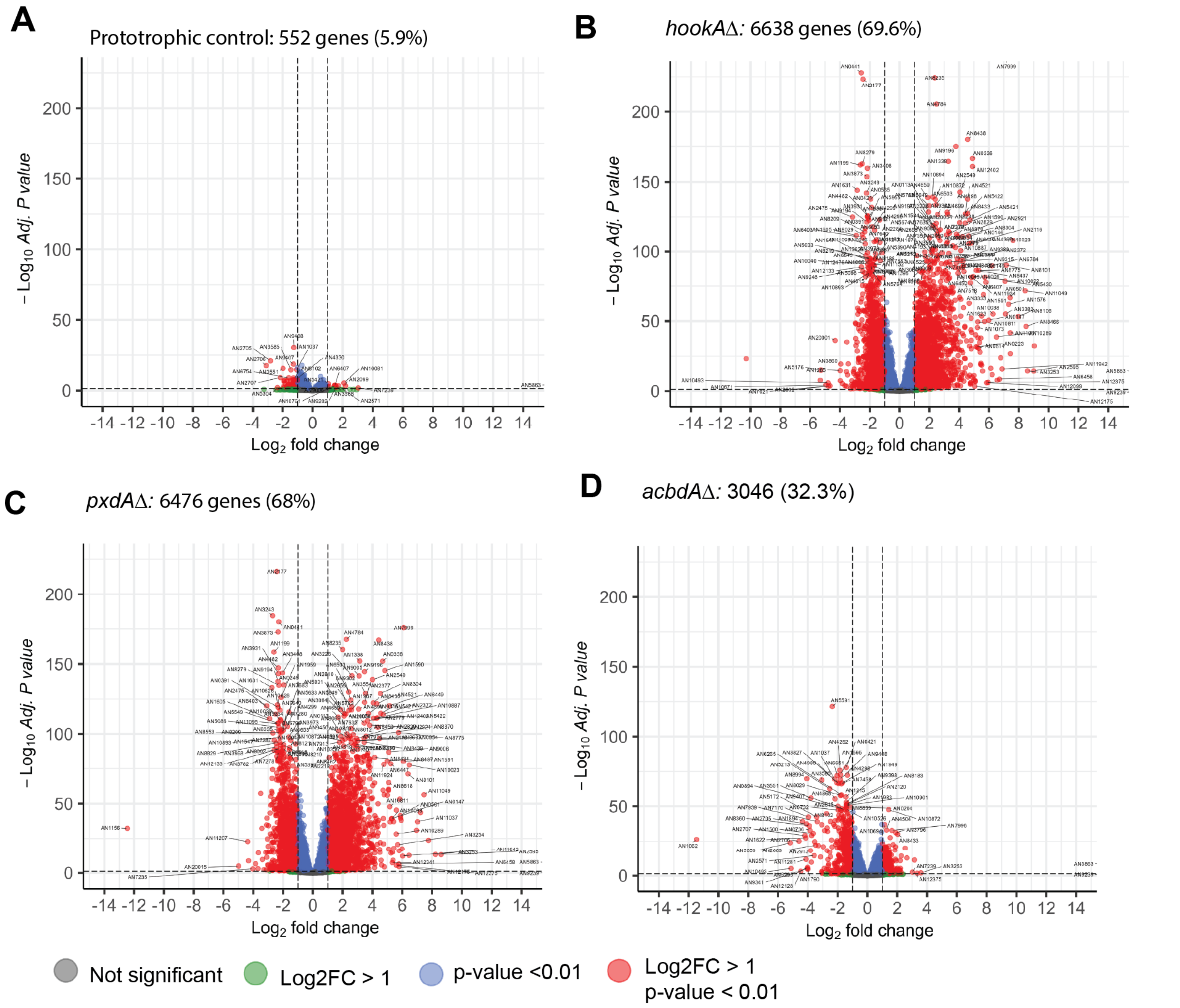

Figure S2 (related to figure 2). (A-D) Volcano plots summarizing the Log2 fold changes in gene expression and adjusted p-values in prototrophic control, *hookAΔ*, *pxdAΔ*, and *acbdAΔ* compared to wild type strain**.** Parenthetical values represent the percentage of detected transcripts that were significantly differentially expressed.

bioRxiv preprint doi: https://doi.org/10.64898/2026.03.03.708097; this version posted March 4, 2026. The copyright holder for this preprint (which was not certified by peer review) is the author/funder, who has granted bioRxiv a license to display the preprint in perpetuity. It is made available under aCC-BY 4.0 International license.

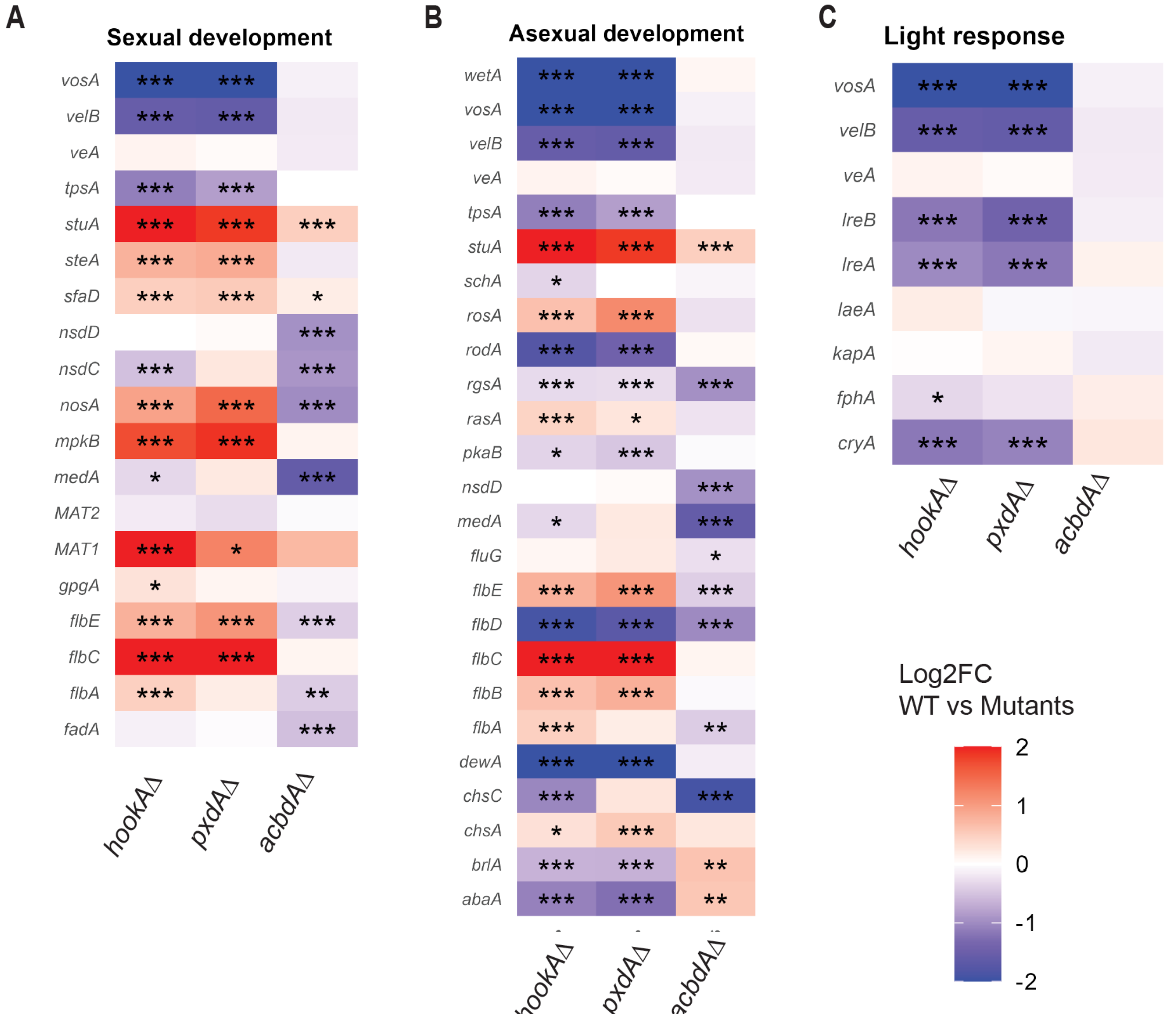

Figure S3 (related to figure 2). Developmental pathway genes are differentially expressed in *hookAΔ* and *pxdAΔ* (A-C) Heatmaps of standardized transcript levels of mutants as compared to the WT for essential reproductive genes involved in asexual development (A), sexual development (B), and light response (C). Data are represented as mean ± SD (n = 4 biological replicates). “ * $p < 0.05$, ** $p < 0.01$, *** $p < 0.001$, **** $p < 0.0001$ and n.s (not significant) by two-way ANOVA.”

bioRxiv preprint doi: https://doi.org/10.64898/2026.03.03.708097; this version posted March 4, 2026. The copyright holder for this preprint (which was not certified by peer review) is the author/funder, who has granted bioRxiv a license to display the preprint in perpetuity. It is made available under aCC-BY 4.0 International license.

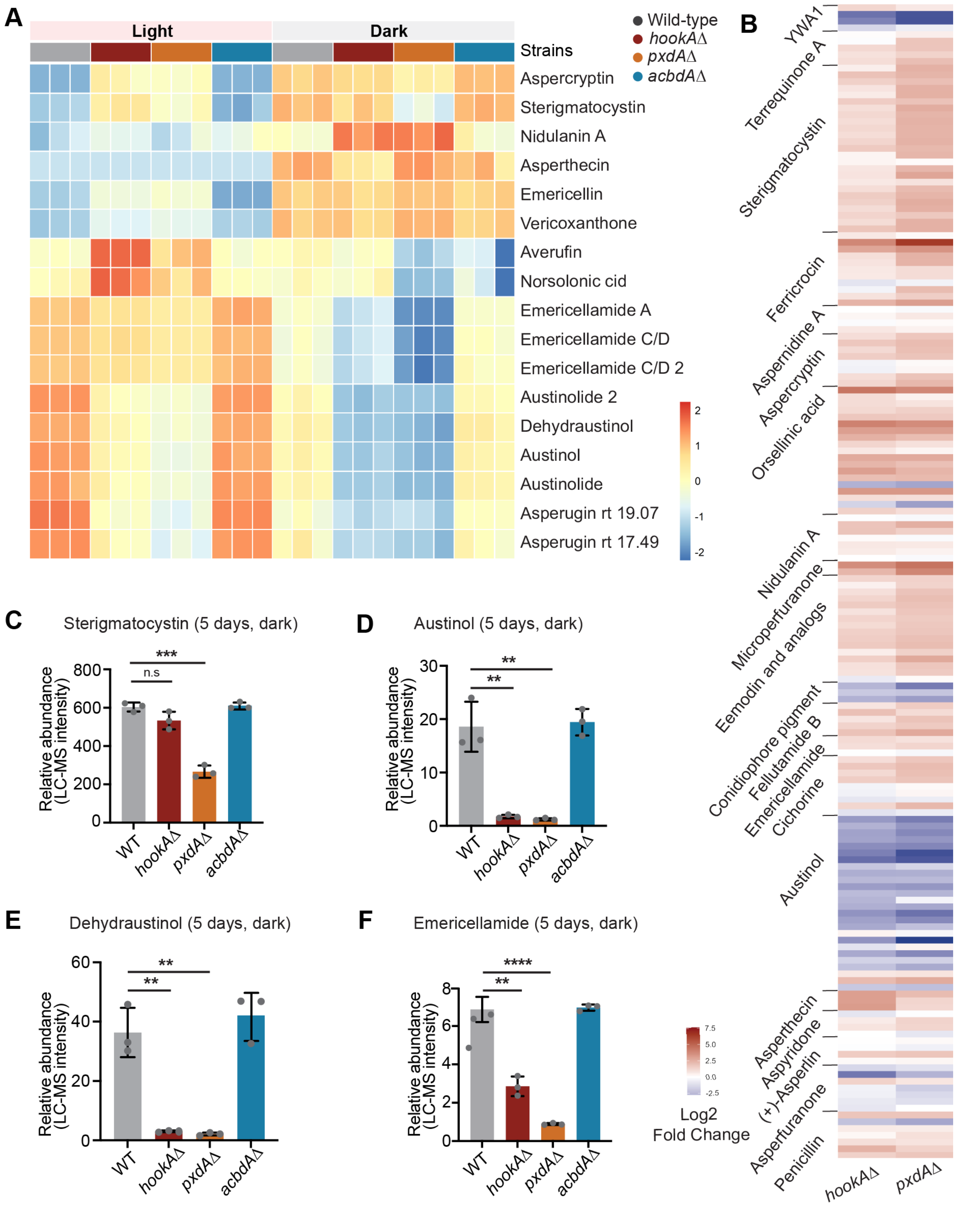

Figure S4 (related to figure 4). Loss of *pxdA*, *hookA*, and *acbdA* alters secondary metabolite gene expression and metabolite production in *Aspergillus nidulans*. (A) Heatmap showing levels of differentially regulated secondary metabolites in WT, *pxdAΔ*, *hookAΔ*, and *acbdAΔ* strains grown under light and dark conditions for five days. The color scale represents high (red) and low (blue) abundance values detected by LC–MS analysis. **(B)** Heatmap showing log_2 fold changes in gene expression between mutants and wild type grown in the dark for five days, covering all secondary metabolism genes from well-characterized clusters with known products. **(C–F)** Relative abundance plots of sterigmatocystin, austinol, dehydroaustinol, and emericellamide in WT, *hookAΔ*, *pxdAΔ*, and *acbdAΔ* strains grown in the dark for five days. Data are represented as mean ± SD (n = 3 biological replicates). " ** $p < 0.01$, *** $p < 0.001$, **** $p < 0.0001$, n.s (not significant) by unpaired t-test."

bioRxiv preprint doi: https://doi.org/10.64898/2026.03.03.708097; this version posted March 4, 2026. The copyright holder for this preprint (which was not certified by peer review) is the author/funder, who has granted bioRxiv a license to display the preprint in perpetuity. It is made available under aCC-BY 4.0 International license.

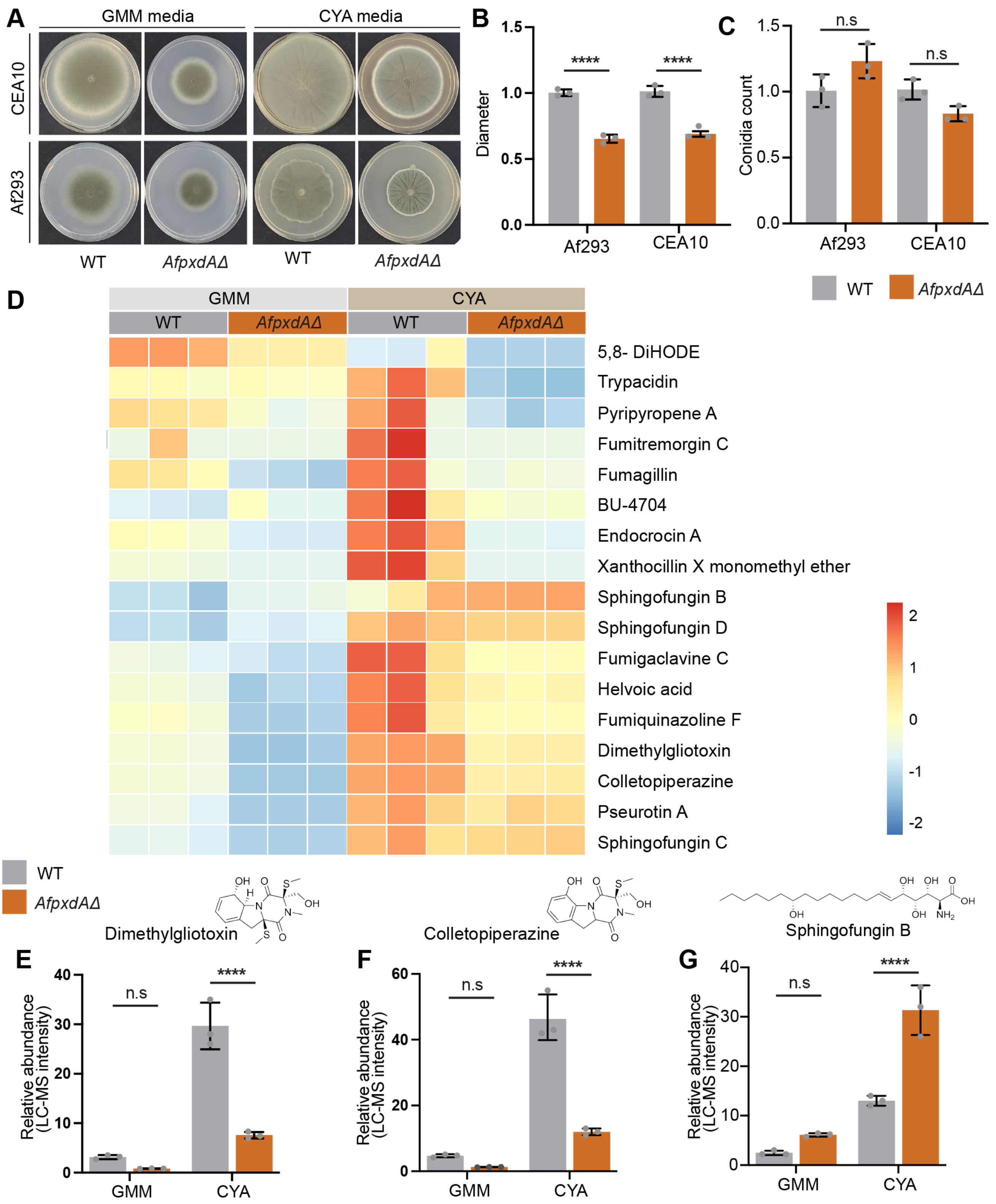

Figure S5 (related to figure 5). ***Loss of PxdA and HookA effects secondary metabolite production in A. fumigatus:*** **(A)** Image of agar plate showing growth phenotype for *A. fumaigatus (*CEA10 and Af293) wild -type and *AfpxdΔ* strains grown on glucose minimal media (GMM) and Czapek yeast extract agar (CYA) media under dark conditions. **(B)** Quantification of radial growth on GMM plates normalized to WT strain. Data are represented as mean ± SD (n = 4 biological replicates). **(C)** Quantification of conidia from the strains grown on GMM plates and count was normalized to the WT strain. **(D)** Heatmap showing levels of differentially regulated secondary metabolites in Af293 WT and *AfpxdΔ*, strains grown on GMM and CYA media under dark conditions at 25°C. The color scale represents high (red) and low (blue) abundance values detected by LC–MS analysis. **(E-F)** Bar plots showing the relative abundance of dimethylgliotoxin (E), colletropiperazine (F), in Af293 WT and *AfpxdΔ* strains. **(G)** Bar plots showing the relative abundance of sphingofungin B in CEA10 WT and *pxdAfΔ* strains. Data are represented as mean ± SD (n = 3 biological replicates). " **** $p < 0.0001$ and n.s. (not significant) by two-way ANOVA."

bioRxiv preprint doi: https://doi.org/10.64898/2026.03.03.708097; this version posted March 4, 2026. The copyright holder for this preprint (which was not certified by peer review) is the author/funder, who has granted bioRxiv a license to display the preprint in perpetuity. It is made available under aCC-BY 4.0 International license.

Table S1: Strains used in this study

Strain	Genotype	Source
RPA1492 (SJR2)	*A. nidulans*: pyrG89, pyroA4, nkuA :: bar, veA$^+$	Gift from Richard Fischer lab
RPA1608	*A. nidulans:* [mGFP-acuE :: AfpyrG], pyrG89, pyroA4, nkuA :: bar, veA$^+$	This study
RPA1627	*A. nidulans:* [mGFP-acuE :: AfpyrG], pyrG89, pxdA-mKate :: Afpyro], pyroA4, nkuA :: bar, veA$^+$	This study
RPA1732	*A. nidulans:* [mGFP-acuE :: AfpyrG], pyrG89, [pxdA*Δ* :: Afpyro], pyroA4, nkuA :: bar, veA$^+$	This study
RPA1736	*A. nidulans:* [mGFP-acuE :: AfpyrG], pyrG89, [hookA*Δ* :: Afpyro], pyroA4, nkuA :: bar, veA$^+$	This study
RPA1756	*A. nidulans:* [mGFP-acuE :: AfpyrG], pyrG89, [acbdA*Δ* :: Afpyro], pyroA4, nkuA :: bar, veA$^+$	This study
RPA1832	*A. fumigatus: Af293, ΔnkuA::mluc, pyrG-*	Lim et al. 2018[86]
RPA1777	*A. fumigatus: Af293, ΔnkuA::mluc, pyrG-, AfpyrG*	Lim et al. 2018[86]
RPA1778	*A. fumigatus: Af293, [pxdAΔ :: parapyrG], ΔnkuA::mluc*	This study
RPA1780	*A. fumigatus: CEA10, ku80Δ*	Ferreira ME et al. 2006[87]
RPA1781	*A. fumigatus: CEA10, [pxdAΔ :: parapyrG], ku80Δ*	This study
RPA1833	*A. fumigatus: CEA10, ku80Δ, pyrg-*	Ferreira ME et al. 2006[87]

Table S2: Plasmids used in this study

Plasmid name	Construct name	Source
RPB2047	*(mGFP-acuE :: AfpyrG)*	This study
RPB990	*(pxdA-mKate :: Afpyro)*	Salogiannis et al. 2016[10]
RPB2646	*(pxdAΔ :: Afpyro)*	This study
RPB2400	*(hookAΔ :: Afpyro)*	This study
RPB2853	*(acbdAΔ :: Afpyro)*	This study

bioRxiv preprint doi: https://doi.org/10.64898/2026.03.03.708097; this version posted March 4, 2026. The copyright holder for this preprint (which was not certified by peer review) is the author/funder, who has granted bioRxiv a license to display the preprint in perpetuity. It is made available under aCC-BY 4.0 International license.

Table S3: Primers used in this study

**Primer name**	**Sequence**	**Source**
mGFP-AcuE- Target DNA F	TAGGAGCTTGGAAGACAAGG	This study
mGFP-acuE- Target DNA R	CTTCTGGACGTCCTATTCG	This study
acbdA-KO Target DNA F	TTGCTATTTCTGATCCTGGGAATCCTG	This study
acbdA-KO Target DNA R	TGACCATGATTACGCCAAGC	This study
pxdA-KO Target DNA F	AGTGACACGGAAGGTTGGTCAATC	This study
pxdA-KO Target DNA R	GACGTTGACACTTCGTGCTAGAACT	This study
hookA-KO Target DNA F	CATGCTTGCTTCCTCTTGCGCTCGC	Songster et al. 2023[16]
hookA-KO Target DNA R	AATAACTGTTGAAGGAGATCCTGAC	Songster et al. 2023[16]
pxdA-mKate Target DNA F	CCTTCAGGAGCGAGTGGCGCATCTC	Salogiannis et al. 2016[10]
pxdA-mKate Target DNA R	GACGTTGACACTTCGTGCTAGAACT	Salogiannis et al. 2016[10]
pxdA-KO-5' F	AGAGGCCATCGCTTTTCTCCCC	This study
pxdA-KO-5' R	CGATATCAAGCTTATCGATACCGTCGA	This study
	CATACCCGCGAACTGACTTAAAGACCAAG	
A. parasiticus pyrG F	GTCGACGGTATCGATAAGCTTG	Calvo et al. 2004[74]
A. parasiticus pyrG R	ATTCGACAATCGGAGAGGCTGC	Calvo et al. 2004[74]
pxdA-KO-3' F	GTCGCTGCAGCCTCTCCGATTGTCGAATG	This study
pxdA-KO-3' R	AGGCCACTCGACGTCTCTGAATCTTCC	This study
	GACGCAACAACCTAGATCAGGC	
pxdA-KO confirmation F	TGGCTCCTCGAGGTTTATTCGC	This study
pxdA-KO confirmation R	GCCGCTGCTGGCATTGACATT	This study
